# Consecutive lynestrenol and cross-sex hormone treatment in biological female adolescents with gender dysphoria: a retrospective analysis

**DOI:** 10.1186/s13293-016-0067-9

**Published:** 2016-02-16

**Authors:** Lloyd J. W. Tack, Margarita Craen, Karlien Dhondt, Heidi Vanden Bossche, Jolien Laridaen, Martine Cools

**Affiliations:** Department of Pediatrics and Genetics, Ghent University, Ghent, Belgium; Division of Pediatric Endocrinology, Department of Pediatrics, Ghent University Hospital, Ghent, Belgium; Division of Pediatric Neurology and Metabolism, Department of Pediatrics, Ghent University Hospital, Ghent, Belgium; Division of Child Psychology, Department of Pediatrics, Ghent University Hospital, Ghent, Belgium; Princess Elisabeth Children’s Hospital, Building 3K12D, De Pintelaan 185, 9000 Ghent, Belgium

**Keywords:** Gender dysphoria, Cross-sex hormone treatment, Safety, Transsexualism, Adolescents, Lynestrenol

## Abstract

**Background:**

Prior to the start of cross-sex hormone therapy (CSH), androgenic progestins are often used to induce amenorrhea in female to male (FtM) pubertal adolescents with gender dysphoria (GD). The aim of this single-center study is to report changes in anthropometry, side effects, safety parameters, and hormone levels in a relatively large cohort of FtM adolescents with a diagnosis of GD at Tanner stage B4 or further, who were treated with lynestrenol (Orgametril®) monotherapy and in combination with testosterone esters (Sustanon®).

**Methods:**

A retrospective analysis of clinical and biochemical data obtained during at least 6 months of hormonal treatment in FtM adolescents followed at our adolescent gender clinic since 2010 (*n* = 45) was conducted. McNemar’s test to analyze reported side effects over time was performed. A paired Student’s *t* test or a Wilcoxon signed-ranks test was performed, as appropriate, on anthropometric and biochemical data. For biochemical analyses, all statistical tests were done in comparison with baseline parameters. Patients who were using oral contraceptives (OC) at intake were excluded if a Mann-Whitney *U* test indicated influence of OC.

**Results:**

Metrorrhagia and acne were most pronounced during the first months of monotherapy and combination therapy respectively and decreased thereafter. Headaches, hot flushes, and fatigue were the most reported side effects. Over the course of treatment, an increase in musculature, hemoglobin, hematocrit, creatinine, and liver enzymes was seen, progressively sliding into male reference ranges. Lipid metabolism shifted to an unfavorable high-density lipoprotein (HDL)/low-density lipoprotein (LDL) ratio; glucose metabolism was not affected. Sex hormone-binding globulin (SHBG), total testosterone, and estradiol levels decreased, and free testosterone slightly increased during monotherapy; total and free testosterone increased significantly during combination therapy. Gonadotropins were only fully suppressed during combination therapy. Anti-Müllerian hormone (AMH) remained stable throughout the treatment. Changes occurred in the first 6 months of treatment and remained mostly stable thereafter.

**Conclusions:**

Treatment of FtM gender dysphoric adolescents with lynestrenol monotherapy and in combination with testosterone esters is effective, safe, and inexpensive; however, suppression of gonadotropins is incomplete. Regular blood controls allow screening for unphysiological changes in safety parameters or hormonal levels and for medication abuse.

## Background

Gender dysphoria (GD), formerly referred to as gender identity disorder [1–3], is defined as the discrepancy between the expressed or experienced gender and one’s natal gender, which causes distress or impairment in important areas of functioning. It is increasingly recognized that many transsexual adults have experienced GD already from childhood onwards [4]. The Endocrine Society clinical practice guideline, published in 2009, recommends medical treatment and psychological guidance of children and adolescents with GD [5].

Because GD will persist after puberty in only a minority of the children presenting with GD, medical treatment, i.e., puberty suppression is best started after the first physical signs of puberty [4, 6]. At the onset of puberty, the reaction of the adolescent to the first bodily changes, under the form of increasing aversion of their biological sex which will enhance GD, often provides additional diagnostic evidence [7]. If GD persists, the child will be eligible for medical treatment aimed at suppressing puberty and/or attenuating its physical symptoms [5, 8], a decision which is to be made by an experienced multidisciplinary team. However, delaying puberty is controversial. The general consensus nowadays tends to be that the advantages of reducing psychological burden, giving more time to explore gender identity and decreasing the need for (and extent of) later sex reassignment surgery [4] outweigh the disadvantages. Arguments against puberty suppression include that the gender identity of adolescents is still developing during puberty and suppression of endogenous sex hormones may interfere with normal growth, bone maturation, and brain development. However, with the initiation of cross-sex hormones, these effects are believed to be (mostly) reversible [9].

To suppress endogenous gonadal hormones in female to male (FtM) adolescents and enlighten the psychological burden of menstruation, two treatment options are available: Gonadotropin releasing-hormone analogs (GnRHa) and progestins. Although prospective randomized clinical trials focussing on physical changes, side effects, and psychological outcome have never been performed, GnRHa are often preferred because they more effectively reduce endogenous ovarian hormone production [5]. However, in many countries, expensive GnRHa are not reimbursed to treat GD, and in these cases, androgenic progestins are a valuable alternative to induce amenorrhea, especially in adolescents who already have advanced development of secondary sex characteristics at the start of treatment or in adults [10]. Lynestrenol (L) (Orgametril®) 5 mg is approximately 13 times cheaper than GnRHa (€85 versus €1100 per year in Belgium), and as it is taken orally, it does not require intramuscular injections. Both medications are not reimbursed to treat adolescents with GD in Belgium and are at the expense of patients. For these reasons, L is mostly used in our center to suppress menstruation in FtM adolescents with an established diagnosis of persisting GD at Tanner stage B4 or further who are not eligible yet for cross-sex hormones (CSH) therapy in view of their young age [10].

L is a prodrug that is converted to norethisterone [11]. It is an androgenic, first generation progestin of the 19-nortestosterone steroids family that is commonly used as hormonal replacement therapy in postmenopausal women or to treat endometriosis [12]. Older studies have revealed that 19-nortestosterone derivates moderately decrease serum triglyceride levels and deteriorate glucose tolerance in women with already impaired insulin secretion. In healthy young women however they will not alter glucose metabolism [13, 14]. The induction or increase of acne and hirsutism by androgenic progestins results from reduction of estradiol and sex hormone-binding globulin (SHBG) levels, leading to higher absolute and relative concentrations of endogenous androgens and unbound androgenic progestins [15]. No studies exist on the long-term use of lynestrenol or other progestins for the treatment of FtM gender dysphoric adolescents.

In our center, CSH under the form of testosterone esters (TE), Sustanon® is added to the treatment from 16 years onwards if GD persists and if the adolescent increasingly lives in the male gender role. This age criterion, although recommended by the Endocrine Society guidelines in 2009 [5], is actually under debate and will likely be adjusted in a revised version [5], which is currently in preparation. Most importantly, the decision to start CSH is made by the multidisciplinary team, in accordance with the adolescent and her/his parents. Increasing doses of intramuscular TE are administered according to a strict protocol, starting with an initial dose of 50 mg every 2 weeks. Every 6 months the dose is increased with 25 mg until an average adult dose of 125 mg per 2 weeks is reached. Between the ages of 17 and 19, an initial dose of 100 mg TE per 2 weeks is given, which is increased to 125 mg after 6 months. After having reached an adult replacement dose, long-acting testosterone undecanoate injections (Nebido®) are often considered more convenient.

The effects of TE administration have been well studied in adults. It will increase facial and body hair, libido, muscle mass, and the oiliness of the skin. It will also result in clitoromegaly, a deeper voice, cessation of menses, redistribution of fat mass, and in some cases male pattern balding [5, 16-19]. Although testosterone administration may cause hypercholesterolemia, hypertension, and reduced high density lipoprotein (HDL) levels, there is no evidence that this increases cardiovascular pathology in FtM transsexuals [20]. During treatment, bone mass density will be maintained because of aromatisation of testosterone to estradiol [21, 22]. Although rare, induction of hormone-related cancers such as carcinomas of the female genital tract and breasts due to testosterone administration has been reported [23, 24]. The use of TE in adolescents with GD specifically has not been studied. It is hypothesized that the same bodily changes, side effects, and hormonal shifts occur as in adults. It was shown recently that TE from 16 years onwards can only partially reverse the decline in bone mass density observed during puberty suppression with GnRHa [25,26].

The aim of this single-center study is to retrospectively analyze the impact of consecutive treatment with L monotherapy and in combination with TE on physical characteristics, safety, metabolic parameters, and hormone levels in a relatively large cohort of FtM gender dysphoric adolescents and to report side effects that occurred during this treatment.

## Methods

### Patients

Data on 45 gender dysphoric FtM adolescents who had received hormonal treatment over a period of at least 6 months from 2010 until September 2015 were available; two adolescents were excluded: one had committed suicide during the follow-up period, and the family was not contacted to obtain informed consent, and one did not consent in use of his data for the study. In five of the remaining 43 cases, insufficient laboratory data were available; therefore, only anthropometric data were included. In some of the remaining 38, due to the retrospective nature of the study and occasional sample loss, not all parameters were available at each time point.

Treatment consisted of L monotherapy for at least 6 months in all included participants followed by combination therapy of L and TE (L+T) in a subset of them (*n* = 25) for at least 6 months. The others were too young to be eligible for CSH therapy at the time of data analysis. Criteria to start CSH therapy were based on the Endocrine Society guidelines [5]. Adolescents who had low vitamin D levels were advised to take vitamin D supplementation and a calcium enriched diet during treatment.

### Methods

Intake visits were aimed at excluding a disorder of sex development underlying GD and at determining the pubertal (Tanner) stage. L was started in FtM adolescents with Tanner stage B4 and further, who met the criteria as outlined in the Endocrine Society guidelines [5]. TE were added to the treatment according to the protocol represented in Table 1, in FtM adolescents of at least 16 years old who met the criteria as outlined in the Endocrine Society guidelines [5]. Follow-up visits were scheduled every 6 months. At each visit, the following parameters were recorded:

**Table 1:** Schedule of increments of testosterone administration and vitamin D supplementation

Schedule 16 years				Schedule 17–19 years			
Time	Substance	Dose	DI	Time	Substance	Dose	DI
Start	Sustanon	50 mg	Every 2 w	Start	Sustanon	100 mg	Every 2 w
6 m	Sustanon	75 mg	Every 2 w	6 m	Sustanon	125 mg	Every 2 w
12 m	Sustanon	100 mg	Every 2 w	12 m	Sustanon	125 mg	Every 2 w
18 m	Sustanon	125 mg	Every 2 w	18 m	Sustanon	125 mg	Every 2 w

+Vit D 25,000 U every 4 w oral + calcium intake of 1200–1500 mg/day

*m* months, *w* weeks, *DI* dose interval

- *Medical history* including family and personal medical history, life style factors (such as smoking habits and alcohol consumption), psychiatric comorbidity, and effects and side effects of the medication. Patients were clearly instructed that in case of metrorrhagia, they should double the L dose for 10 days
- *Physical examination*: anthropometry, blood pressure, Tanner stage, acne, and hirsutism
- *Biochemical analyses*: every 6 months: complete blood count, electrolytes, liver and renal function, thyroid-stimulating hormone (TSH), free thyroxin (fT4), luteinizing hormone (LH), follicular stimulating hormone (FSH), estradiol (E2), total and free testosterone (T and fT), and sex hormone-binding globuline (SHBG). Every year: fasting glucose, insulin, lipid metabolism, and anti-Müllerian hormone (AMH). During medical treatment, patients are seen every 3 months by the team child psychologist. In the absence of psychiatric comorbidity, they are evaluated twice by the team child psychiatrist during this phase; once before initiation of L and once more at start of L+T. Fertility issues are discussed thoroughly throughout each treatment phase, and adolescents are given the chance to undergo ovum pick-up following an ovarian stimulation program before initiation of T. However, most—if not all—adolescents prefer to start TE without any delay. When considering sex reassignment surgery, adolescents are again given the above option or, alternatively, part of their ovaries can be cryopreserved at the time of gonadectomy. In our experience, most patients prefer the second option.

Statistical analysis was performed using IBM SPSS software package (version 22). A *P* value of less or equal to 0.05 was considered significant. McNemar’s test for comparison of paired data was performed to analyze reported side effects over time. After testing for normality, anthropometric and biochemical data were analyzed using a paired Student’s *t* test or a Wilcoxon signed-ranks test as appropriate. For biochemical analyses, all statistical tests were done in comparison with baseline parameters (at start of L or L+T). Eight patients were using oral contraceptives (OC) at intake. Data obtained in these patients at intake were excluded from analyses if a Mann-Whitney *U* test indicated influence of OC.

Methods of measurements of the biochemical parameters are represented in Table 4. The detection limit for LH, E2, and T (RIA) was 0.1 U/L, 25 ng/L, and 10 ng/dL, respectively. The maximal detection limit for SHBG was 200 nmol/L. In case of values below or above these limits, the limit itself was inputted for statistical analyses.

Approval of the ethics committee of Ghent University hospital was obtained (B670201525328). All patients were contacted by mail and could object against use of their data for the study.

## Results

### Patient and treatment characteristics

Data on educational level, comorbidities, and lifestyle characteristics are represented in Table 2.

**Table 2:** Summary of patient characteristics and side effects

Comorbidity	Education	Side effects			
Psychiatric: 11/43 (25.6 %) Social problems: 7/43 (16.3 %) DSD: 0/43 (0 %)	ASO: 9/42 (21.4 %) TSO: 16/42 (38.1 %) BSO: 11/42 (26.2 %) BUSO: 4/42 (9.5 %) KSO: 2/42 (4.8 %)	Time	Metrorrhagia	Acne	
		L0 L6m L12m L + T0 L + T6m L + T12m	– 19/39 (48.7 %) 5/28 (17.9 %) 4/25 (16.0 %) 5/22 (22.7 %) 4/16 (25.0 %)	6/41 (14.6 %) 10/39 (25.6 %) 8/28 (28.6 %) 6/25 (24.0 %) 13/22 (59.1 %) 6/16 (37.5 %)	
Smoking	Alcohol	Time	Headache	Hot flushes	fatigue
No: 34/43 (79.1 %) Moderate: 9/43 (20.9 %) High: 0/43 (0 %)	No: 24/43 (55.8 %) Yes: 19/43 (44.2 %)	L L + T	5/41 (12.1 %) 0/25 (0 %)	4/41 (9.8 %) 0/25 (0 %)	3/41 (7.3 %) 2/25 (8 %)

Smoking: Moderate: 1 to 20 cigarettes a day, high: >20 cigarettes a day

*ASO* theoretical education, *TSO* technical education, *BSO* vocational training, *BUSO* school for children with learning difficulties, *KSO* art school, *L* lynestrenol monotherapy, *L+T* lynestrenol and testosterone esters combination therapy

Mean age at start of L and L+T was 15 years 10 months, and 17 years 5 months, respectively. Mean treatment duration for L was 12.6 months and for L+T 11.4 months. No patients stopped treatment because they no longer wished to pursue gender reassignment.

### Side effects

Reported side effect is shown in Table 2. Headaches and hot flushes were reported during L monotherapy, whereas fatigue was a complaint during both L and L + T. One of the four patients with hot flushes stopped treatment because of this side effect. During L, there was a non-significant increase in acne (*P* = 0.125); however, the prevalence of acne significantly increased in the first 6 months of L+T (*P* = 0.021), requiring treatment with oral retinoic acid in three out of 13 individuals. Metrorrhagia was mainly reported in the first 6 months of L but significantly dropped in the next 6 months (*P* = 0.004). During L+T, the prevalence of metrorrhagia increased slightly over the course of treatment.

### Anthropometry

Mean height at start of L was 164.6 cm, and at start of L+T, it was 167.6 cm. Weight and body mass index (BMI) significantly increased in the first 6 months (*P* = 0.004 and *P* = 0.031, respectively), but had turned back to baseline after 12 months of L (*P* = 0.538 and *P* = 0.918, respectively). L+T was associated with a significant and continuous weight gain after 6 months (*P* = 0.023 and *P* = 0.003) and 12 months (*P* = 0.002 and *P* = 0.015, respectively). This increase in weight and BMI was significantly different from weight changes in age-matched same biological sex peers, based on standard deviation (SD) scores [27]. Evolution of weight and BMI are represented in Table 3.

**Table 3:** Summary of analysis of antropometric data

	L0	L6m	*P (L0-6 m)*	L12m	*P (L0–12 m)*	L+T0	L+T6m	*P (L+T0–6 m)*	L+T12m	*P (L+T0–12 m)*
Weight	61.48	63.98	*0.004*	61.03	*0.007* ^a^	58.65	61.18	*0.023*	65.10	*0.001*
SD	0.16	0.36	*0.120*	0.01	*0.911*	−0.38	−0.13	*0.016*	0.01	*0.001*
BMI	22.58	23.00	*0.031*	22.39	*0.193* ^b^	20.69	22.38	*0.003*	23.26	*0.004*
SD	0.21	0.43	*0.098*	0.18	*0.719*	−0.34	0.20	*0.011*	0.24	*0.004*

*SD* standard deviation in comparison with Flemish peers [27], *L0* mean values before initiation of L, *L6m* mean values after 6 months of L; *P(L0–6 m) P* values of comparison of baseline parameters with values after 6 months of L, *L12m* mean values after 12 months of L, *P(L0–12 m) P* values of comparison of baseline parameters with values after 12 months of L, *L+T0* mean values before initiation of L+T, *L+T6m* mean values after 6 months of L+T, *P(L+T0–6 m) P* values of comparison of baseline parameters with values after 6 months of L+T, *L+T12m* mean values after 12 months of L+T, *P(L+T0–12 m) P* values of comparison of baseline parameters with values after 12 months of L+T, *L* lynestrenol monotherapy, *L+T* lynestrenol and testosterone esters combination therapy

^a^Although mean weight of all patients seems to decrease, a significant increase in weight was noted when comparing weight at baseline with weight after 12 months of L. This discrepancy is caused by exclusion of patients, who had not yet reached 12 months of L at time of analysis

^b^BMI non-significantly increased after 12 months of L, although mean BMI seems to decrease. This is due by the same phenomenon as described in a

### Biochemical analyses

#### Safety and metabolic parameters

**Fig. 1:**
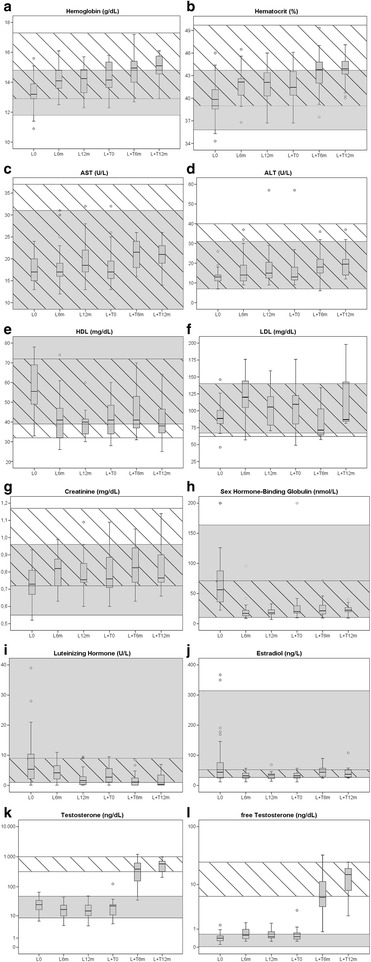
Box-and-whisker plots of biochemical parameters. *L0* baseline values, *L6m* after 6 months of L, *L12m* after 12 m of L, *L+T0* before start of L+T, *L+T6m* after 6 months of L+T, *L+T12m* after 12 months of L+T. **a** Hemoglobin (g/dL, multiply by 10 for SI units: g/L); **b** hematocrit (%, multiply by 0,01 for SI units: proportion of 1.0); **c** AST (U/L, multiply by 0.0167 for SI units: μkat/L); **d** ALT (U/L, multiply by 0.0167 for SI units: μkat/L); **e** HDL (mg/dL, multiply by 0.0259 for SI units: mmol/L); **f** LDL (mg/dL, multiply by 0.0259 for SI units: mmol/L); **g** creatinine (mg/dL, multiply by 88.4 for SI units: μmol/L); **h** sex hormone-binding globulin (nmol/L); **i** luteinizing hormone (U/L); **j** estradiol (ng/L, multiply by 3.671 for SI units: pmol/L); **k** testosterone (ng/dL, multiply by 0.0347 for SI units: nmol/L); **l** free testosterone (ng/dL, multiply by 34.7 for SI units: pmol/L). *L * lynestrenol monotherapy; *L+T* lynestrenol and testosterone esters combination therapy, *AST/ALT* aspartate/alanine amino transferase, *HDL/LDL* high/low density lipoprotein

Mean hemoglobin (Hb) and hematocrit (Hct) levels increased significantly in the first 6 months of L and of L+T but remained stable in the next 6 months. None of the individual Hb values rose above the upper adult male reference (Fig. 1a, b).

Only alanine amino transferase (ALT) but not aspartate amino transferase (AST) showed a statistically significant, although not clinically relevant rise after 12 months of L. In one patient, ALT levels transiently increased above the upper male reference to 57 U/L after 12 months of L but normalized after the start of L+T. Both ALT and AST further increased under L+T treatment but remained well within the male reference range. None of the patients reached the threshold of three times the upper reference limit which we considered the cutoff to stop treatment (Fig. 1c, d). Creatinine significantly increased during the first 6 months of L and during the first 6 months of L+T but remained stable in the following 6 months (Fig. 1g).

Total cholesterol and triglyceride levels did not change during treatment; however, mean HDL decreased significantly and mean low-density lipoprotein (LDL) levels increased significantly in the first 6 months of L. During L+T, mean LDL levels did not change significantly (Fig. 1e, f). No significant changes in hemoglobin A1c (HbA1c), glucose levels, insulin levels, or homeostasis model assessment (HOMA) index were noticed during either L or L+T treatment.

#### Hormone levels

Although no significant changes in mean TSH levels were observed, fT4 levels increased significantly both in the first and second half year of L. In the first 6 months of L+T, there was a decrease in TSH accompanied with a significant and consecutive decrease in fT4 in the first and next 6 months of treatment. However, in all patients, serum levels for both TSH and fT4 remained well within the reference range (Table 4). The eight patients who were using OC before L was started were excluded for baseline analysis of LH, FSH, E2, A, and SHBG. Mean AMH, T, and fT levels were not different in OC users as compared to non-OC users and were included in baseline analyses.

**Table 4:** Summary of the analysis of biochemical data

Test Method of measurement	L0	L6m	*P (L0–6 m)*	L12m	*P (L0–12 m)*	L+T0	L+T6m	*P (L+T0–6 m)*	L+T12m	*P (L+T0–12 m)*
Hemoglobin Spectrophotometry (Sysmex XE-5000)	133.7	142.4	<0.001	141.4	0.001	143.3	148.5	<0.001	149.7	<0.001
	Reference (g/L): M < 18 y: 130–160, M > 18 y: 129–173, F < 18 y: 120–160, F > 18 y: 118–148									
Hematocrit DC impendance (Sysmex XE-5000)	0.400	0.417	<0.001	0.418	0.003	0.419	0.435	0.002	0.438	<0.001
	Reference (proportion of 1.0): M < 18 y: 0.37–0.49, M > 18 y: 0.39–0.497, F < 18 y: 0.36–0.46, F > 18 y: 0.358–0.437									
Creatinine Rate-blanked Jaffé kinetic assay (Roche Diagnostics c701 (a + b))	65.416	71.604	<0.001	70.72	<0.001	69.836	73.372	0.052	72.488	0.045
	Reference (μmol/L): M/F: 11–13 y: 46.852–69.836, M/F: 13–15 y: 50.388–76.908, M > 15 y: 63.648–103.428, F > 15 y: 48.62–84.864									
Aspartate amino transferase UV-kinetic (IFCC) method without pyridoxal phosphate (Roche Diagnostics Cobas c701)	0.30	0.31	0.903	0.33	0.091	0.31	0.35	0.031	0.35	0.003
	Reference (μkat/L): M 0–0.62, F 0–0.52									
Alanine amino transferase UV-kinetic (IFCC) method without pyridoxal phosphate (Roche Diagnostics Cobas c701)	0.22	0.27	0.121	0.31	0.012	0.28	0.31	0.079	0.34	0.045
	Reference (μkat/L): M 0.12–0.67, F 0.12–0.52									
Triglycerides Enzymatic colorimetric method (GPO-PAP and CHOD-PAP, Roche Diagnostics c701 (a + b))	0.838	0.870	0.31	0.661	0.128	0.651	0.934	0.18	1.394	0.18
	Reference (mmol/L): M 10–15 y: 0.362–1.413, M 15–20 y: 0.418–1.672, F 10–15 y: 0.418–1.48; F 15–20 y: 0.441–1.492									
Total cholesterol Enzymatic colorimetric method (GPO-PAP and CHOD-PAP, Roche Diagnostics c701 (a + b))	4.153	4.348	0.182	4.237	0.218	4.212	4.099	0.504	4.450	0.11
	Reference (mmol/L): M 10–15 y: 3.082–5.232, M 15–20 y: 2.927–5.102, F 10–15 y: 3.212–5.206, F 15–20 y: 3.108–5.258									
High density lipoprotein Enzymatic colorimetric method (Roche Diagnostics c701 (a + b))	1.481	1.096	<0.001	1.017	0.002	1.098	1.194	0.419	1.085	0.77
	Reference (mmol/L): M: 0.829–1.865, F: 1.010–2.486									
Low density lipoprotein Calculated	2.379	3.057	0.001	2.750	0.043	2.794	2.267	NA	3.163	0.09
	Reference (mmol/L): M < 20 y: 1.606–3.626, F < 20 y: 1.735–3.626									
Hemoglobin A1c Ion-exchange chromatography (Tosoh HLV-723G8)	0.052	0.051	0.228	0.052	0.34	0.051	0.052	0.102	0.051	0.317
	Reference (proportion of 1.0): M/F 0.04–0.055									
Homeostasis Model Assessment insulin resistance Calculated	2.99	3.11	0.122	2.43	0.396	2.45	4.84	NA	7.44	0.185
	Reference: M during puberty <5.22, F during puberty <3.82 or M/F <4.39 [55, 56]									
Thyroid-stimulating hormone Electro-chemoluminescence assay (Roche Diagnostics E170 Modular)	2.07	2.06	0.757	2.1	0.257	2.25	1.83	0.013	2.22	0.271
	Reference (mIU/L): M/F 11–20 y: 0.51–4.3									
Free thyroxin Electro-chemoluminescence assay (Roche Diagnostics E170 Modular)	15.959	17.375	0.006	19.820	<0.001	18.275	16.216	0.001	14.543	0.003
	Reference (pmol/L): M/F 12-20 y: 12.613-20.978									
Lutheinizing hormone Electro-chemoluminescence assay (Roche Diagnostics E170 Modular)	7.56	4.63	0.065	2.58	0.042	3.41	1.93	0.004	1.68	0.028
	Reference (IU/L): M 1–9 U/L, F 1–96 U/L (cycle dependant)									
Follicular-stimulating hormone Electro-chemoluminescence assay (Roche Diagnostics E170 Modular)	5.15	5.18	0.785	4.36	0.623	4.96	2.95	0.001	2.56	0.019
	Reference (IU/L): M 1–12 U/L, F 2–22 U/L (cycle dependant)									
Sex hormone-binding globulin Electro-chemoluminescence assay (Roche Diagnostics E170 Modular)	77.14	20.88	<0.001	19.15	0.001	30.34	25.27	0.279	23.51	0.279
	Reference (nmol/L): M < 70 y: 11.6–71.2, F < 50 y: 10.5–163.7									
Estradiol Electro-chemoluminescence assay (Roche Diagnostics E170 Modular)	277.564	119.895	0.002	120.225	0.122	117.802	175.841	0.107	156.348	0.701
	Reference (pmol/L): M 99.484–191.626, F 98.016–1152.694 (cycle dependant)									
Testosterone liquid chromatography tandem mass spectrometry (LC/MSMS)	0.950	0.667	0.002	0.663	0.687	0.844	15.559	<0.001	19.532	0.001
	Reference (nmol/L): M 11.139–34.874, F < 50 y 0.291–1.669									
Free testosterone Calculated	15.962	24.290	0.209	21.861	0.138	25.678	295.297	0.005	472.614	0.008
	Reference (pmol/L): M 208.2–867.5, F 0.694–22.208									
Anti-Müllerian hormone Enzyme-linked immunosorbent assay (Beckman Coulter Company) until 2/2015, thereafter electro-chemoluminescence assay (Roche Diagnostics E170 Modular)	33.214	25.714	0.066	24.000	0.423	24.357	28.429	0.18	27.072	0.575
	Reference (pmol/L): M 6–20 y 11.429–1028.578 (Tanner), F 8–20 y: 4.7143–60.143									

*L0* mean values before initiation of L, *L6m* mean values after 6 months of L, *P(L0–6 m) P* values of comparison of baseline parameters with values after 6 months of L, *L12m* mean values after 12 months of L, *P(L0–12 m) P* values of comparison of baseline parameters with values after 12 months of L, *L+T0* mean values before initiation of L+T, *L+T6m* mean values after 6 months of L+T, *P(L+T0–6 m) P* values of comparison of baseline parameters with values after 6 months of L+T, *L+T12m* mean values after 12 months of L+T, *P(L+T0–12 m) P* values of comparison of baseline parameters with values after 12 months of L+T, *NA* not available due to insufficient data, *L* lynestrenol monotherapy, *L+T* lynestrenol and testosterone esters combination therapy, *M* male reference, *F* female reference, *Y* years old, *cycle dependant* different reference ranges according to different stages of menstrual cycle (maximum upper and lower limit of all cycle stages are represented), *Tanner* different reference ranges according to different Tanner stages (maximum upper and lower limit of all Tanner stages are represented)

Mean SHBG, LH, but not FSH levels decreased sharply during the first 6 months of L and remained unchanged in the next 6 months (Fig. 1h). Only after L+T, LH and FSH were both fully suppressed (Fig. 1i). L caused a significant decrease in mean E2 levels at 6 months with no significant changes anymore thereafter (Fig. 1j). Mean AMH levels did not change during the course of treatment.

The significant decrease in T levels in the first 6 months of L was accompanied by a non-significant increase in fT . Both T and fT did not change in the next 6 months. As expected, mean T increased significantly in the first months of L+T, already at the lowest dose (50 mg/2 weeks) and further increased in the next months to reach T values well within the male reference range. This was accompanied by a similar increase in fT levels. Some patients exceeded the male upper reference of 25 ng/dL, due to blood sampling close to the last TE injection (Table 4 and Fig. 1k,l).

## Discussion

Androgenic progestins are a cheap alternative for GnRHa to suppress menses in gender dysphoric FtM adolescents. They are therefore preferred in situations where GnRHa are not reimbursed, especially in older adolescents who already have advanced pubertal development at the time of diagnosis, either as monotherapy while the adolescent is awaiting eligibility (depending on the local team’s criteria) to start cross-sex hormone treatment or in combination with the latter to reinforce its effects. Since no data are available on the safety profile and effects of progestins in general and L in particular for this indication, we analyzed these parameters retrospectively in a relatively large cohort of FtM adolescents treated in our center between 2010 and 2015. Our study population did not differ from the adolescent Belgian population in terms of educational level, smoking habits, and alcohol consumption [28–30].

The most frequently reported *side effects* were metrorrhagia (almost 50 % after 6 months of L) and acne (almost 60 % after 6 months of L + T but also prevalent during L). In many but not all cases, metrorrhagia was limited and could be controlled by doubling the L dose during 10 days; metrorrhagia tends to be less prevalent with longer treatment duration. An increase in acne in women during androgenic progestins or androgen administration is a well-known phenomenon [10, 17, 18, 31, 32]. However, adolescents are particularly vulnerable for this side effect. In 3/13 (23.1 %) of adolescents with acne during L+T, vitamin A analogs were required. Combining L+T with vitamin A analogs did not lead to exacerbation of liver enzymes or important changes in other safety parameters.

Consecutive L and L+T treatment does not seem to interfere with residual growth. In contrast, weight and BMI significantly increased during L+T as compared to age-matched same biological sex peers, which is most likely due to changes in lean body mass, as is seen in athletes who use androgenic anabolic steroids [32]. Longitudinal standardized assessment of body composition is necessary to confirm this hypothesis.

Analysis of *safety parameters* was mostly reassuring, and no patients had to stop treatment because of an adverse safety profile: Throughout the treatment, hemoglobin, hematocrit, and creatinine shifted into, but did not exceed, the male reference range. Indeed, androgens are known to stimulate erythropoiesis, renal erythropoietin production [31, 33], and muscle mass [34]. Similarly, liver enzymes increased during L+T but remained well within the male reference range in all patients. Since androgens have been shown to (transiently) elevate liver enzymes, rarely causing severe liver disease [32, 35–39], we advise close monitoring during treatment. Consistent with other studies [13, 14, 31], no changes in HbA1c, insulin, glucose, or HOMA index were found during the entire course of treatment. Importantly, our treatment regimen resulted in a more unfavorable lipid profile. Similar findings have been reported, mostly in adults [40, 41]. However, there are currently no data available on the metabolic profile and cardiovascular risk in older adult transmen who changed their gender during adolescence [25]; this finding merits attention, and further research in transsexual adults focusing on early determinants of cardiovascular disease such as adiponectin or carotid artery intima media thickness is warranted.

The expected *hormonal changes* of L were obvious after 6 months of monotherapy: T had decreased by almost 30 % whereas E2 had decreased by almost 60 %. The overall decrease in the estrogenic to androgenic ratio is a common property of all androgenic progestins [15, 40, 42]. Similarly, cross-sex hormone therapy resulted in mean T levels within the male reference range already after 6 months and with the lowest doses of 50 mg T per 2 weeks only. The non-significant rise in E2 levels during L+T likely represents the effect of aromatisation of the injected testosterone esters.

LH, but not FSH, was only partially suppressed by L monotherapy. Complete suppression of both gonadotropins was only achieved during L+T.

(Androgenic) progestins have been shown to reduce TBG levels, resulting in an increase in fT4 [43], which was also observed in our study. During L+T, TSH transiently and fT4 persistently decreased. The impact of sex steroids on thyroid function is poorly understood and various studies have yielded conflicting data [44, 45]. Overall, changes in TSH and fT4 were small in our study and did not result in clinical or biochemical hypo- or hyperthyroidism. Therefore, we did not consider them as clinically relevant.

Whether or not long-term androgen exposure in natal women alters the ovarian follicle reserve, limiting the possibilities for successful ovarian cryopreservation and subsequent in vitro follicle maturation, is currently debated. In primates, androgen administration has been shown to stimulate early follicular growth, after which further development is stopped due to suppression of gonadotropin secretion, resulting in an ovarian morphology similar to polycystic ovary (PCO) syndrome and increased AMH levels [46–49]. Similar PCO-like changes have been observed in ovaries of transmen after salpingo-oophorectomy; however, this was not confirmed in a more recent study [50–53]. In contrast with the study of Caanen et al. [54] where AMH levels were strongly reduced using a combination of T, an aromatase inhibitor, and a GnRHa to treat GD in adult natal women, AMH levels did not significantly change in our patients. Further clinical and pathological studies are needed to examine the impact of androgen treatment on AMH levels and ovarian morphology and follicle reserve in natal women with GD.

Our study has the typical limitations of a retrospective analysis, such as a number of missing data and the impossibility to draw causal relationships. Reported side effects were limited to those recorded in the patient’s files and can therefore be an underestimation. Strengths of our study are the relatively large and homogenous patient population and the fact that this is a single center study where all patients were treated by only two different observers and received the same treatment regimen and follow-up schedule according to a strict protocol. It is, to our knowledge, the first report on the effects of L/progesteron treatment in FtM adolescents with GD and one of the few studies reporting on CSH treatment in GD adolescents.

## Conclusions

This study shows that treatment of FtM adolescents with L effectively and significantly decreases the overall estrogenic to androgenic ratio within 6 months and that it can be used as a safe and cheap alternative to GnRHa to suppress menses. However, although no direct comparison has been made, it is probably less effective than GnRHa in inducing total amenorrhea and in suppressing gonadotropins and hence development of secondary sex characteristics. Therefore, L is specifically indicated in adolescents with advanced pubertal development and in situations where GnRHa are not reimbursed, to reduce psychological burden while they are awaiting eligibility for cross-sex hormone treatment.

## Abbreviations

ALT: alanine amino transferase
AMH: anti-Müllerian hormone
AST: aspartate amino transferase
Crea: creatinine
CSH: cross-sex hormones
E2: estradiol
FSH: follicular stimulating hormone
GD: gender dysphoria
GnRHa: gonadotropin-releasing hormone analogs
fT: free testosterone
fT4: free thyroxin
Hb: hemoglobin
HbA1c: hemoglobin A1c
Hct: hematocrit
HDL: high-density lipoprotein
HOMA: homeostasis model assessment
L: lynestrenol
LH: luteinizing hormone
LDL: low-density lipoprotein
OC: oral contraceptives
SHBG: sex hormone-binding globulin
T: testosterone
Tchol: total cholesterol
TE: testosterone esters
